# Resource allocation across the dementia continuum: a mixed methods study examining decision making on optimal dementia care among health and social care professionals

**DOI:** 10.1186/s12913-021-06230-9

**Published:** 2021-03-18

**Authors:** Fiona Keogh, Tom Pierse, David Challis, Eamon O’Shea

**Affiliations:** 1 Centre for Economic and Social Research on Dementia, National University of Ireland Galway, Newcastle Road, Galway, H91 TK33 Ireland; 2 Institute of Mental Health, University of Nottingham, Nottingham, NG7 2RD UK

**Keywords:** Dementia, Balance of care, Decision making, Resource allocation

## Abstract

**Background:**

The understanding of appropriate or optimal care is particularly important for dementia, characterised by multiple, long-term, changing needs and the increasing expectations of people using services. However, the response of health and social care services is limited by resource constraints in most countries. This study sought to determine the optimal level, mix and cost of services for different dementia case types across the dementia continuum, and to better understand the resource allocation decision making process among health and social care professionals (HSCPs).

**Methods:**

A balance of care framework was applied to the study questions and developed in three ways; firstly by considering optimality across the course of dementia and not just at the margin with residential care; secondly, through the introduction of a fixed budget to reveal constrained optimisation strategies; and thirdly through the use of a mixed methods design whereby qualitative data was collected at workshops using nominal group technique and analysed to obtain a more detailed understanding of the decision-making process. Twenty four HSCPs from a variety of disciplines participated in the resource allocation decision-making exercise.

**Results:**

HSCPs differentiated between case type severity; providing 2.6 times more resources to case types with higher level needs than those with lower level needs. When a resource constraint was introduced there was no evidence of any disproportionate rationing of services on the basis of need, i.e. more severe case types were not favoured over less severe case types. However, the fiscal constraint led to a much greater focus on meeting physical and clinical dependency needs through conventional social care provision. There was less emphasis on day care and psychosocial provision when resources were scarcer following the introduction of a fixed budget constraint.

**Conclusions:**

HSCPs completed complex resource allocation exercises for people with dementia, including expected differentiation across case type severity. When rationing was introduced, HSCPs did not discriminate in favour of case types with high levels of need. They did, however, support conventional home care provision over psychosocial care, although participants were still keen to provide some residual cover for the latter, especially for case types that might benefit.

**Supplementary Information:**

The online version contains supplementary material available at 10.1186/s12913-021-06230-9.

## Introduction

Dementia is acknowledged as a significant health challenge globally [1, 2]. The scale of the challenge is not simply due to the numbers, but because people with dementia require a wide range of health and social care services to address their physical, psychological and emotional needs, over potentially a long time period of time [3]. Most people with dementia live at home, with family caregivers providing the bulk of care [4]. Therefore, the needs of informal caregivers and the person’s living circumstances also need to be factored into the overall demand on the health and social care system.

Public sector expenditure on existing service provision for dementia care accounts for an estimated €1.7bn or 52% of the total cost of dementia in Ireland [5], a similar proportion to that reported in other jurisdictions [4, 6]. Estimated expenditure on community based services for people with dementia in 2018 was €138 m, with an average annual expenditure per person with dementia of €3862 [7]. Home support hours accounted for the bulk of this expenditure; less than 1% of the €138 m was spent on psychosocial supports. Service provision for dementia is characterised as geographically variable with a low level of provision in relation to need as measured by dementia prevalence [7]. In general, spending in Ireland on community based services and home care in particular is recognised as being too low to meet current levels of need [8].

Expectations around dementia have changed, due in part to the work of Kitwood [9] and others. Dementia has been reframed from a medical condition to be managed, to a psychosocial narrative, such that people with dementia and others talk about ‘living well with dementia’ and proactively supporting personhood as part of a wider quality of life agenda [10–12]. Informed by the disability rights movement, there are increasing demands for individualised services and supports that address the needs of the whole person [13]. Individualised services recognise the person’s life experience and support the person to remain at home, maintaining their abilities, interests and social contacts for as long as possible, rather than their entering residential care prematurely because of the absence of adequate alternative services [14–16]. A social model of dementia care is increasingly referenced in policy both nationally and internationally. The Irish National Dementia Strategy is based on the principles of personhood and citizenship. However, this policy rhetoric is not matched by resource allocation decision-making as referenced above [17]. At the practice level, defining and assessing personhood-related needs is difficult. So too is devising an optimal care plan that must be delivered within budget. Although they can be cost-effective, newer forms of service provision are likely to add to existing costs unless these are used as substitutes to more traditional forms of provision [18].

In the context of demographic changes, increasingly expensive health and care services and budgetary constraints, there has been some research focus on identifying the optimal mix of community and institutional long-term care for older people and for people with dementia [18–20]. Conceptually, the balance of care (BoC) approach has been concerned with identifying optimum care for a patient group on the margin between different care settings, typically older people on the margin of home/community and residential care [21]. However, the issue of optimality arises *throughout* the dementia journey which has a deteriorating trajectory, often with multiple identifiable ‘boundary’ or transition points [22].

The RightTimePlaceCare study identified four factors which were particularly important in determining who could be diverted from institutional care: costs/finances; individual circumstances/care needs; informal care availability and formal care availability [20]. However, there is little understanding of how individual decision-makers in the health and social care community balance these factors in making resource allocation decisions, and how these decisions are likely to change over the dementia continuum. Are different factors given different weight in early stage dementia for people with milder symptoms, compared to factors which are important at later stages for people with more advanced symptoms? In the context of constrained resources for health and social care, how are different types of service provision prioritised given the wider array of services and supports that are required and expected? How important is psychosocial provision for people with dementia at various points on the dementia continuum? More generally, what constitutes ‘optimal care’ for different case types, not just those at the boundary between home and residential care but more widely, particularly as new models of care for people with dementia are becoming more important in the policy process.

The understanding of appropriate or optimal care is particularly important for dementia, characterised by multiple, long-term, changing needs and the increasing expectations of people who need services, within a wider context of resource constrained health and social care services. The objective of this study was to explore the views of health and social care professionals (HSCPs) on current and optimal resource allocation for different dementia case types, with and without fixed budget constraints. An expanded balance of care framework and mixed methods design was used to explore optimality across the course of dementia and not just at the margin with residential care, incorporating the introduction of a fixed budget constraint to reveal implicit service priorities.

## Method

The study design and methods were informed by policy makers in a partnership process embedded in the research funding and by people with dementia using a patient and public involvement (PPI) approach. A detailed analysis of qualitative data generated by the research has already been reported, covering the general themes influencing allocation deliberations and the adoption of decision-making heuristics by participants [23]. Building on a BoC framework, we have added an economic dimension in this paper through examining decisions under two scenarios; an ‘optimal needs-led scenario’, hereinafter described as no constraint (NC) scenario and a budget constrained (BC) scenario, where people had to make decisions under a fixed budget constraint. Using a mixed methods design, the quantitative data generated by the BoC method is presented alongside relevant and newly reported qualitative data to gain a more detailed understanding of the resource allocation decision-making process among HSCPs under the two budget scenarios.

### Participants

HSCPs from a range of disciplines, who had direct experience of working with, or allocating services to people with dementia were identified from four health regions in Ireland. An information sheet describing the study was sent to these individuals, along with an invitation to participate in one of five workshops that were organised around the country. Twenty nine HSCPs were invited and five could not participate due to scheduling conflicts. Twenty four attended including; public health nurses (PHNs) (*n* = 6), social workers (*n* = 3), occupational therapists (OT) (*n* = 2), physiotherapists (*n* = 1), speech and language therapists (SLT) (n = 1), dieticians (n = 1), psychologists (n = 1), mental health nurses (n = 2), home care coordinators (*n* = 4) and older person’s service managers (n = 3). People with dementia and family carers took part in separate workshops and their findings are reported separately [24].

### Research design

**Fig. 1:**
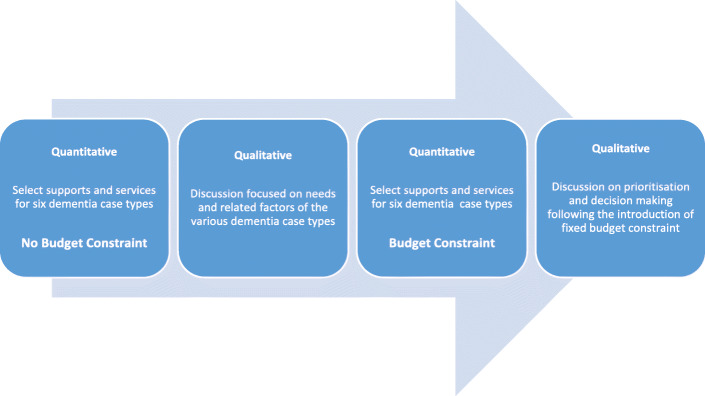
Research design

The study used an explanatory sequential design with qualitative phases following on from quantitative phases as shown in Fig. 1 [25]. Varied order sequencing was not used because it was impractical.

Nominal group technique (NGT) was used to structure the quantitative exercises and qualitative discussion in five workshops, each with a multidisciplinary mix of HSCPs. The NGT method is used for exploring healthcare priorities as it facilitates equal participation [26]. The materials used were vignettes to illustrate six case types and a service list.

### Development of case types and vignettes

Case types were developed using the approach adopted by Challis et al. [19]. As is the case in many jurisdictions, there is no single data set available in Ireland containing all of the required variables to generate potential dementia case types, so data from several sources were combined. The main data set used to construct dementia case types was 277 anonymised home care assessments for people with dementia [27]. A sub-set of six dementia case types, comprising 46% of the dementia case types in the dataset was then selected for the study (Table 1). A dataset of anonymised InterRai assessments for 453 inpatients over 65 [28] provided additional data on level of cognitive impairment which was not recorded in the home care assessment data. The InterRai single assessment is a comprehensive IT based standardised assessment used to assess the health and social care needs of people. Further descriptive variables were sourced from the literature and assigned to the case types based on reported prevalence, specifically behavioural and psychological symptoms of dementia [29] and comorbidities [30] and attitude to specific care services among people with dementia. Study participants reported that each of the dementia case types used in the study was credible: “*with all of them [case types] I could think of a patient with that”* [HSCP CHO9]. Vignettes were developed for each dementia case type to lend realism and to help participants consider the needs of each case in allocating services. A sample vignette is included in supplementary material.

**Table 1:** Variables and data sets used in the development of the case types

	Data set: Assessments for home care service for people with dementia (*N* = 277)							Variables derived from Single Assessment Tool data (*N* = 453 inpatients) and literature			
Case Type	Dependency (Low, Medium, High)	Falls risk (Y/N)	Communication difficulty (Y/N)	Living alone (Y/N)	% of cases	Age	Sex (M/F)	Cognition (Mild, Moderate, Severe)	BPSD	Comorbidity	Amount of informal support (Low, Medium, High)
1	Low	N	N	N	9.5	84	F	Mild	None	Hypertension, Diabetes	Low
2	Medium	N	N	N	5.0	79	M	Mild	Depression and anxiety	Coronary Heart Disease	Medium
3	Medium	N	N	Y	9.5	82	F	Moderate	Irritability and persecution	Hypertension	Medium
4	Medium	Y	N	Y	9.9	86	M	Moderate	Wandering and hallucinations	None	Low
5	Medium	Y	Y	Y	3.4	83	F	Severe	Apathy and sleeping problems	Stroke	Medium
6	High	Y	Y	N	8.4	80	F	Severe	Sleep problems and psychotic symptoms	Hearing loss	High

### Service types and costs

Participants were asked to allocate services as appropriate to needs across the six dementia case types. They were given a service list which was based on that used by Giebel et al. [31] modified for the Irish context and informed by a mapping study of dementia-specific services in Ireland carried out in 2016/17 [32]. Twenty community-based service types were listed (see Table 2).

**Table 2:** Service List

Service	
Home Care	
In-home Respite/Sitting Service (known as ‘visiting service’)	
Re-ablement / Dementia support worker	
Day Care (Standard or Dementia Specific)	
Alzheimer’s Café, Dementia Social Clubs, or other support group for people with dementia	
Dementia Friendly Activities	
Meals on Wheels	
Transport	
Dementia Advisor	
Carer Education Programme	
Dementia Carer Support Groups	
Counselling for family carer	
Dementia Cognitive Therapies	
Public Health Nurse	
Specialist Dementia/Case Management	
Day Hospital (Primary Care Centre)	
Physiotherapist	
Occupational therapist	
Other Primary Care (Speech and Language/Dietician/Hearing)	
Aids and Appliances (basic)	
Referral to Psychiatry of Old Age Team	
Nursing home based respite	
Nursing Home Bed	

Unit costs for these services were calculated based on Irish Health Service Executive staff pay scales and the Irish literature on unit costs [18]. The full service cost was used irrespective of the funding source, so that service prioritisation could be compared on a like for like basis. The full cost of a Psychiatry of Old Age referral and a carer education programme were included in the budget allocation for the hypothetical month, although in practice this cost may be spread over a longer period. Further detail on the costing of services is available in the supplementary material.

### Workshops

A three hour workshop was designed to collect the qualitative and quantitative data. Two exercises were run: one with no budget constraint (NC) and one with a budget constraint (BC). The six vignettes, the service list and service definitions were provided to participants, and a computer, pre-loaded with a spreadsheet showing the list of potential services that could be allocated for each dementia case type. Unit costs were embedded in the spreadsheet, but were hidden for the first NC exercise. The pilot study and data from a recent national audit of services used by people with dementia in Ireland were used to derive the monthly budget constraint [7]. Five workshops were held and each was audio recorded.

#### NC scenario

In this scenario, participants were asked to allocate the type and amount of services that would be of most benefit to the person and carer in each of the six vignettes without considering budget constraints. Participants in the workshop then, in turn, presented to the group their allocation rationale for the case types, focusing on the needs they were trying to address through the service allocation.

#### BC scenario

In this scenario, the costs of the services allocated in the first scenario for each case type were revealed. Participants were instructed to do the same exercise again but to work within an overall budget constraint of €7000 to allocate care for all six dementia case types for 1 month. Although participants felt that this level of expenditure approximately reflected the current availability of resources, many found it difficult to stay within this constraint and tended to ‘overspend’. It was not feasible to enforce the constraint rigidly in the first three workshops and the average budget de facto expanded to €8928, 28% above the initial constraint. For the final workshop, the budget was increased to €10,000 per month across the six dementia case types to explore whether a more relaxed constraint made the exercise easier for participants to complete.

In the BC scenario, time was allocated for discussion as to which services participants decided to cut in order to meet the budget constraint, and why, with an emphasis on articulating their decision making process. Finally, the average budget allocated per dementia case type was calculated and displayed and the group discussed whether they agreed with the overall and relative allocations or if they wanted to make any changes after seeing the various relativities. This constituted a consensus check which allowed participants to review and recalibrate their choices if required.

### Data analysis

The data from the workshops was compiled from the spread sheet workbooks. The type and amount of services allocated for each dementia case type for both the NC and BC scenarios were compiled. For the budget constrained scenario, all participant information was included irrespective of whether the person achieved the target budget level. In situations where an individual recommended nursing home placement, this allocation was not included in the averaging of community services.

For the qualitative data analysis all recordings were transcribed and uploaded to NVivo version 12. The six-phase method of thematic analysis [33] was used to analyse the data using a general inductive approach [34]. The design of the study resulted in qualitative data that was quite structured and the general inductive approach allowed us to identify the core meanings relevant to the research objectives. A detailed description of the qualitative analysis and findings is in Keogh et al. [23]. This qualitative analysis identified five themes: (1) whose needs are being met; (2) what needs are identified; (3) decision making context; (4) decision making process; and (5) allocation outcomes. A framework was developed to illustrate the relationships among themes. For example, the first three themes influenced theme 4, the decision making process. The decision making process resulted in allocation outcomes (theme 5) which included not just services, but the identification of key roles and functions. In order to explore the effect of the resource constraint on decision making in the allocation of resources, further examination of relationships within data across the themes was conducted, resulting in the identification of five ‘decision rules’ or heuristics that were used by the HSCPs when faced with the budget constraint.

The heuristics can be summarised as follows:

- Heuristic 1: with constrained resources, supports for the person with dementia are prioritised. Supports for the carer are focused on maintaining their ability to continue caring for the person with dementia.
- Heuristic 2: with constrained resources, personal care and clinical needs of the person, and carer burden are prioritised.
- Heuristic 3: with constrained resources, proactive or preventive care for the person with dementia and the carer, and psychosocial needs for both are not prioritised.
- Heuristic 4: with constrained resources a limited number of personal context factors are considered – those that pose a risk and those that most directly relate to the person and/or impinge on the ability of the carer to provide support are to the fore.
- Heuristic 5: in general, decision-makers need as much knowledge about the person and their circumstances as possible, to tailor the optimum support package for this person at this point in time and to avoid under or over-provision.

### Stakeholder engagement, public and patient participation (PPI)

As part of an applied partnership study, the research questions were identified in collaboration with senior managers from the National Dementia Office and national managers for older person’s services in Ireland. The study used public and patient involvement (PPI) methods to involve people with dementia. A person with dementia was a member of the Oversight Group for this study, with input into study design, methods and measures. The list of services and case type vignettes were developed in consultation with two further people with dementia and two carers of people with dementia [23].

#### Findings

Table 3 shows that the monthly allocation of resources in the unconstrained scenario is 2.6 times higher than current fixed budget allocations; €25,421 relative to €9684. The aggregate funding by dementia case type, showed a range of average total monthly spend in the NC scenario of €2136 to €5706 and a range of €765 to €2034 in the BC scenario. Both scenarios showed a gradient in spending, with the highest resource use evident in case types 4, 5 and 6 (highest needs) and the lowest in case 1 (lowest needs). In allocating resources, HSCPs were differentiating between need at some global level; greater need led to higher levels of provision.

**Table 3:** Average total monthly cost per case type and proportion of overall cost for no constraint (NC) and budget constraint (BC) scenario

	Case 1 Low needs	Case 2	Case 3	Case 4	Case 5	Case 6	Total High needs
**No constraint (NC) scenario**							
Total cost of care package	€2136	€2604	€4250	€5203	€5524	€5706	€25,421
Proportion of overall cost	**8.4%**	**10.2%**	**16.7%**	**20.5%**	**21.7%**	**22.4%**	**100%**
**Budget constraint (BC) scenario**							
Total cost of care package	€765	€1149	€1824	€2034	€1913	€1999	€9684
Proportion of Total Cost	**7.9%**	**11.9%**	**18.8%**	**21.0%**	**19.8%**	**20.6%**	**100%**
**Percentage difference in total cost of care package between the two** scenario**s**	**64%**	**56%**	**57%**	**61%**	**65%**	**65%**	**62%**

Following the introduction of the budget constraints, total spend for the six case types was reduced by 62% from that generated by the unconstrained budget. The expenditure for case types 2, 3 and 4 was cut the least, while case types 1, 5 and 6 were cut the most, although the differences were not significant. Participants did not change the distribution of spending across the case types following the introduction of a fixed budget constraint. Instead, they sought to reduce expenditure vertically within dementia case types rather than horizontally ration across case types by withdrawing resources entirely from case types with lower needs. The proportion of the overall budget allocated to each case remained largely the same in both budget scenarios, with a variation between the two of zero to 4 percentage points. It is striking, for example, that case type 1, the lowest level of need, was allocated approximately 8% of the overall budget in each scenario. Case type 1 continued to receive resources, even when fixed budgets were introduced. Thus, it appeared that participants were more focussed upon concerns of equity and the potential of using the full continuum of care, rather than targeting higher levels of need. The qualitative data sheds more light on this. In particular, for those with lower needs, HSCPs were keen to use an array of services to maintain ability for the person with dementia and to support family carers.

Table 4 shows the aggregate funding by service type in both budget scenarios and the proportion of funding that was allocated to services within each scenario. The proportion allocated to home support increased in the constrained scenario from 46 to 49%. Day care showed the largest decrease in allocation from 18% in the NC scenario to 8.6% in the BC scenario. The absolute difference in spending on day care between the two scenarios was 82%. The proportion of funding allocated to psychosocial supports and carer supports was largely the same in both scenarios. However, this conceals the fact that psychosocial provision fell by 69%, second only to day care in terms of the extent of the cutback. Support for carers was also affected. For example, dementia case 5 was allocated 6 weeks of respite in the NC scenario, reduced to 1 week in the BC scenario.

**Table 4:** Differences in funding for service types in each scenario – amount and percentage of allocation within each scenario

Service	Allocated funding in NC scenario	Allocated funding in BC scenario	Amount of change	% Change
**Home support**	€11,807	€4755	-€7053	− 59.7%
	(46.4%)	(49.1%)		
**Day care**	€4569	€833	-€3736	−81.8%
	(18.0%)	(8.6%)		
**Psycho-social support**	€1569	€493	-€1076	−68.6%
	(6.2%)	(5.1%)		
**Visits from Health and Social Care Professionals**	€1320	€522	-€798	−60.5%
	(5.2%)	(5.4%)		
**Carer supports**	€2210	€776	-€1649	−64.9%
	(8.7%)	(8.0%)		
**Other services**	€3946	€2306	-€1640	−41.6%
	(15.5%)	(23.8%)		
**Total spend for six cases**	€25,421	€9684	-€15,737	−61.9%
	(100.0%)	(100.0%)		

The case by case comparison in Table 5 sheds more light on the extent to which HSCPs were attempting to meet specific needs, following the introduction of a fixed budget constraint. For most services, there was little difference in the proportion of funding allocated in the BC scenario compared to the NC scenario. The exception was day care, where every case showed a substantial reduction in the proportion allocated in the BC scenario. In general, the reduced expenditure on day care was largely allocated towards more home care or more carer support, depending on the case type. This was reflected in the importance the participants placed on a preventive approach, through the provision of early intervention and proactive carer support. The latter recognised the dual needs of carers, to maintain their wellbeing but also to provide practical support as family carers enable the person to stay at home:

“There is a huge burden of care really on his family, which is, I feel, wouldn’t be sustainable long term. So to try and prevent family burn out, it would be better to initiate home support services earlier, rather than later.” (HSCP, CHO3)

**Table 5:** Allocation of services by case type and percentages of total case cost in the two scenarios

Service type	Case 1 Lower needs	Case 2	Case 3	Case 4	Case 5	Case 6 Higher needs
**Support in the home** (Home help, support worker and In home respite)						
No constraint scenario	31%	53%	47%	50%	51%	41%
Budget constraint scenario	36%	53%	47%	43%	54%	56%
**Day Care**						
No constraint scenario	20%	6%	17%	17%	18%	24%
Budget constraint scenario	9%	0%	8%	14%	11%	7%
**Psycho-social support** (social clubs and activities, dementia cognitive therapies)						
No constraint scenario	15%	11%	7%	4%	4%	5%
Budget constraint scenario	18%	11%	6%	3%	3%	1%
**Visits from HSCPs** (OTs, Physiotherapists, SLTs, Dieticians, PHNs, Dementia advisers)						
No constraint scenario	6%	5%	4%	5%	5%	6%
Budget constraint scenario	8%	6%	4%	4%	6%	6%
**Carer Supports** (Support groups, counselling, education, nursing home based respite)						
No constraint scenario	7%	7%	8%	6%	11%	12%
Budget constraint scenario	9%	10%	10%	4%	10%	7%
**Other Services**: **Referral to Psychiatry of Old Age Team**						
No constraint scenario	5%	17%	12%	9%	7%	9%
Budget constraint scenario	5%	18%	22%	18%	13%	14%
**Meals on wheels and transport**						
No constraint scenario	17%	1%	4%	10%	5%	3%
Budget constraint scenario	16%	3%	3%	14%	3%	9%

“ … if I could support them [the carers], then they would continue to care for people at home so they wouldn’t need to go to a nursing home.” (HSCP, CHO3)

The opportunity to provide proactive support and maintain the person’s ability was emphasised in the NC scenario, as well as a focus on quality of life such as supports to maintain social contact:

“ … to prevent it, people who are starting to go into … [losing ability] if they could just maintain their social [contact], like doing their shopping with support, going out, having their lunch, interacting, going to the banks with support.” (HSCP, CHO2).

HSCPs reluctantly reduced higher order ‘quality of life’ inputs for more basic levels of care and support at home when faced with cutbacks. The ‘optimum’ service profile for the various case types when there were no constraints included services chosen to enhance the quality of life for the person and carer, as well as services which focused on early intervention and the prevention of difficulties downstream. In the budget constrained scenario, however, services supporting the person’s quality life were sacrificed somewhat to again maintain home care for essential personal care needs and to support the carer as much as possible:

“Cutting out the likes of day care, reminiscence therapy, all the extras and just focusing on like survival, like Meals on Wheels, Home Help, if somebody can’t get out of bed, you know?” (HSCP CHO2)

“Anywhere where there was, you know, where the carer burden was low, I reduced all the in-home respite and things like that because they seemed to be managing, and scraped back to the basics.” (HSCP, CHO2)

This thinking is encapsulated in a decision making heuristic which was identified from the qualitative data; *with constrained resources, proactive or preventive care for the person with dementia and the carer, and psychosocial needs for both are not prioritised* (Heuristic 3) [23]. Allocation decisions thus tended to reflect participants views on recipient dependency and need, including their perceived capacity to benefit from particular services. For example, the profile of services allocated to case type 6, namely proportionately more home support and less day care, psychosocial support and carer support, reflects the high physical dependency of this case, coupled with the lack of ability of the case type to access services such as day care and psychosocial options (see Table 1 for case profiles). Psychosocial provision increased for case type 1, where the potential to benefit from this service was highest, as least as perceived by participants. HSCPs also took into account the stated preferences for care among recipients, for example, the zero allocation to day care in Case 2 was in direct response to the stated preference in that case for no day care. Participants were rationally trying to make decisions on the allocation of scarce resources and employed a number of heuristics to aid in this process 23].

The quantitative findings showed how the HSCPs, when faced with the budget constraint, reduced expenditure vertically within dementia case types rather than horizontally ration across case types by withdrawing resources entirely from case types with lower needs. However, in real life conditions, i.e. in formal service allocation processes, HSCPs noted that the case types with lower needs would receive little or no resource, as observed by one participant; “*and I think if I’m honest, I don’t know would two of those [cases] have got any services, they may just have been waitlisted.”* (HSCP, CHO3).

On completion of the BC exercise, the participants reflected on what they had allocated in the NC scenario, including whether they had ‘misallocated’ to some extent. A common observation was ‘*giving too much because it was available’* and not taking into account the ability of the person/family to actually absorb that much service; *‘there’s lots of people [service providers] going in and out of the house’ (CHO3).* Participants redressed the balance of day care for the cases with higher level of needs, noting that *‘they just wouldn’t be physically able’* for so much day care. The importance of understanding multiple contexts emerged as a key theme in the qualitative analysis and was also captured in a decision making heuristic: *Need as much knowledge about the person and their circumstances as possible, to tailor the optimum support package for this person at this point in time and to avoid under- or over-provision* (Hueristic 5) [23].

In attempting to assess what an ‘optimum’ budget might be for these case types, this might suggest that the total budget in the NC scenario was too high. However, the initial monthly budget of €7000 for all six case types in the BC scenario was deemed too low to maintain carers in their role: “*… they’re just burnt out”* (HSCP CHO3). In addition, as described above, participants were unable to provide any early intervention, preventative care, or services which enhanced quality of life in the BC scenario, and this trade off was made to stay within the constraint.

## Discussion

Overall, this study indicates that HSCPs capably differentiated between case severity in the allocation of resources; the level of service provision is significantly higher for people with the greatest need. In the unconstrained scenario, monthly costs ranged from €2136 in case type 1 to €5706 in case study 6; a similar gradient existed following the introduction of a budget constraint with monthly costs ranging from €765 in case type 1 to €1999 in case study 6. Dementia case type 1 had the lowest needs and received the lowest share of the budget allocation of 8.4 and 7.9% respectively in the unconstrained and constrained scenarios. Dementia case types 4, 5 and 6 were perceived as relatively high need categories and received similar budget shares of approximately one fifth of total resources in both funding scenarios.

The introduction of the budget constraint did not substantially change the range and profile of services that were allocated to each case. Instead providers tried to meet the same broad range of needs, albeit within a much smaller budget – giving less to all service items, but not consistently excluding any of them. Home support accounted for the largest share of the budget in both the constrained and unconstrained scenarios. This is an important finding given the critique of home care offered by self-directed support service models, personal budgets and the debate about their suitability and effectiveness for older people [35–37]. To some extent this reaffirms the importance of ‘traditional’ forms of care such as home support, but home support was also allocated for its potential to meet several needs at once (for example the personal care needs of the personas well as some respite for the carer). However, we have little information as to the effectiveness of home support and whether it is the ‘optimal’ support for the case types presented in this study. The wider availability of alternatives to ‘traditional’ home support, such as enablement or psychosocial support from dementia support workers, could result in these forms being the preferred form of home support and there was some support from the qualitative data for this contention.

The value of psychosocial supports, both in quality of life and cost terms, was strongly endorsed in the qualitative discussions around resource allocation, but still only accounted for around 5% of the budget in both scenarios. Moreover, when the budget constraint was introduced, these services were reduced (except for case type 1), sometimes to such a low level as to be merely tokenistic in terms of potential impact on quality of life and well-being. Some participants recognised the importance of scale and the need to provide an adequate level of psychosocial support in order to have any impact on the day to day life of people with dementia, but their efforts were frustrated by the budget constraint. These findings were evident in the decision making heuristics derived from the qualitative data, for example, heuristic 3 which states that: *with constrained resources, proactive or preventive care for the person with dementia and the carer, and psychosocial needs for both are not prioritised* [23]*.* The importance of psychosocial supports are well documented [1, 38] but in the absence of adequate resourcing, services which are essential to meet personal care needs will inevitably take priority.

The final budget allocation across all case types in the NC scenario was 2.6 times higher than the budget in the BC scenario. This suggests an appreciation and realism about the level of potential unmet need that exists in the system currently, as reflected in the resource allocation decisions of HSCPs when they had unfettered choice. Does this mean that optimum needs-led care for dementia in Ireland should have a budget of up to three time higher than currently exists? Probably not, as the qualitative data indicated different ways in which the participants felt that had *‘over provided’* in their own words in the absence of a budget constraint. They acknowledged it was difficult to resist the temptation to allocate *‘one of everything’* to most case types when there was no budget constraint. Provision in the unconstrained case was acknowledged as potentially being ‘*too much’* for many of the dementia case types and would likely *‘over-burden’* the person/family with service inputs. The sequential nature of the exercise also meant that HSCPs made an initial higher level of provision instead of starting with a lower amount of service and assessing progress as they would typically do in real world conditions. That said, it is clear that services for the various dementia case types are currently under-provided. While optimal allocation may not be at the level provided in the unconstrained scenario, all participants were of the view that the low level of service provided in the constrained scenario was far too low. Social needs were left unmet, particularly those which might improve quality of life (such as social contact) in order to prioritise the absolute minimum of traditional home support for those cases who needed it. These findings were evident in the decision making heuristics derived from the qualitative data, for example, heuristic 2 which states: *with constrained resources, personal care and clinical needs of the person, and carer burden are prioritised* [23].

While the introduction of the budget constraint did not significantly change the profile of services that were allocated to each case, prioritisation did not occur in the way we might have expected. For example, participants chose not to move an allocation completely from one service to another. This did not seem feasible in practice as the case types had diverse needs such as physical dependency, mental health difficulties and a carer who needed support. The other option was to ration across dementia case types, for example, through withdrawing resources from low need case types to higher need case types. It would seem that the principles of equity and a continuum of support were more dominant than focussed targeting of care services in shaping judgements of appropriate resource allocation. Scott et al. [39] describe nurses response to rationing as being informed by an explicit assessment of patient needs, along with ‘often intuitive and implicit prioritisation of care across a group of patients … ’ (p.1532). They note the ethical dilemmas this poses such as the potential for biased or discriminatory decision making, as well as the burden of the personal confrontation with rationing that health care professionals can experience. This was vividly portrayed in the qualitative data from this study where the HSCPs described the difficulty of making allocation decisions under the budget constraint scenario, some finding it upsetting and/or frustrating that they could not, within the given budget, meet the diverse needs of all six cases in this exercise. They were reluctant to neglect any case types under the rationing scenario, continuing to provide support for those with low level need, and, as a by-product, pushing out the budget constraint.

What are the implications of these findings for policy-making in dementia, particularly in regard to resource allocation? The key message is that budgets for people with dementia in Ireland are currently underfunded relative to need, as interpreted by HSCPs. At a minimum, this paper suggests that a relaxation of the budget constraint for home care provision for people with dementia in Ireland is warranted. Furthermore, it would seem that if policy decisions are to target resources upon those with higher levels of need, then the mechanisms to achieve this, such as eligibility criteria, need to be made explicit and be formally endorsed at an organisational level, in order to support the individual practitioner, or front line decision-maker in making potentially difficult decisions. Finally, the preventive potential of the proactive provision of a range of supports along the continuum of dementia will not be realised if psychosocial supports are services are not adequately resourced.

## Conclusions

This mixed methods study examines the decision making process around resource allocation for community dementia services among HSCPs, providing insights into what optimum dementia care might look like for six dementia case types along the dementia continuum. HSCPs completed complex resource allocation exercises for people with dementia, including expected differentiation across case type severity. In a scenario of unconstrained resources they addressed a wide range of needs for both the person with dementia and carer, with an emphasis on personalised care and a preventive approach as recommended in policy. When rationing was introduced, HSCPs did not discriminate in favour of case types with high levels of need. They prioritised conventional home care provision over psychosocial care in order to ensure essential personal care needs were met. Participants were still keen to provide some residual cover psychosocial needs, especially for case types that might benefit. The contrast between the care allocated in the constrained and unconstrained scenarios indicates that budgets for people with dementia in Ireland are currently underfunded relative to need.

### Limitations

Although feedback on the validity of the case types and the list of services was positive, the use of case types and the overall allocation exercise was necessarily artificial in some respects. HSCPs noted that encountering a case in this way and considering the full menu of services for provision was not their usual practice. They would typically see people at a transition or crisis point, which shapes service provision in a certain way and would not have access to such a complete range of services. In particular, HSCPs found having to consider services, which in effect are funded by different budgets, in one single budget, a particular challenge. This may be further complicated by services that fall outside the formal system of care, those that are provided by charitable or voluntary bodies, that do not fall under any health budget or cost code. These type of services, many of which have a psychosocial focus, may be residual to the formal system, leading to their absence from mainstream delivery of care, including the commissioning process. Finally, calculating the units of services in a way that they could be meaningfully allocated within a month was difficult as some services, such as counselling or referral to a psychiatry of old age team, in practice take place over a longer period. This may have led to some skewing of costs but not sufficiently to undermine the findings.

## Supplementary Information

**Additional file 1.** Sample Vignette 4 ‘Mr Dunne’.

## Data Availability

The vignettes used in the study are available in the Zenodo repository at https://zenodo.org/record/4030438#.X708wlX7S70. It was not possible to remove identifying details sufficiently from the qualitative data in this study (focus group and interview transcripts), to ensure the anonymity of the research participants. As a result, this data cannot be made available publicly. The qualitative and quantitative data from the study can be made available for further research upon reasonable request if the research team is assured participants’ anonymity can be protected. To access the data, please contact the corresponding author.

## Abbreviations

BC: Budget Constraint
BoC: Balance of Care
HSCP: Health and Social Care Professional
NC: No budget constraint
NGT: Nominal Group Technique
OT: Occupational Therapist
PHN: Public Health Nurse
PPI: Public and Patient Involvement
SLT: Speech and Language Therapist
