# Supplementary material for: Resource allocation across the dementia continuum: a mixed methods study examining decision making on optimal dementia care among health and social care professionals
Source: BMC Health Serv Res. 2021 Mar 18;21:243. doi: 10.1186/s12913-021-06230-9 (PMC7977590; doi:10.1186/s12913-021-06230-9)
Supplement: Supplementary file 1 — Additional file 1. Sample Vignette 4 ‘Mr Dunne’. [file 12913_2021_6230_MOESM1_ESM.docx]

**Author details**

**Title**: Resource allocation across the dementia continuum: A mixed methods study examining decision making on optimal dementia care among health and social care professionals.

Fiona Keogh: [fiona.keogh@nuigalway.ie](mailto:fiona.keogh@nuigalway.ie), Centre for Economic and Social Research on Dementia, National University of Ireland Galway, Newcastle Road, Galway H91 TK33, Ireland.

Tom Pierse: [tom.pierse@nuigalway.ie](mailto:tom.pierse@nuigalway.ie), Centre for Economic and Social Research on Dementia, National University of Ireland Galway, Newcastle Road, Galway H91 TK33, Ireland.

David Challis: [David.Challis@nottingham.ac.uk](mailto:David.Challis@nottingham.ac.uk), Institute of Mental Health, University of Nottingham, Nottingham NG7 2RD, UK

Eamon O’Shea: [eamon.oshea@nuigalway.ie](mailto:eamon.oshea@nuigalway.ie), Centre for Economic and Social Research on Dementia, National University of Ireland Galway, Newcastle Road, Galway H91 TK33, Ireland.

Corresponding author: Fiona Keogh. Email: fiona.keogh@nuigalway.ie.

**Sample Vignette 4 ‘Mr Dunne’**

**Home Situation**: Mr Dunne is 86 years old and lives alone on a farm in a rural area. The house is heated by an open fire and is in a poor state of repair.

**Activities of Daily Living**: Mr Dunne needs help with eating, dressing and bathing. He often gets confused when dressing and puts his clothes on back to front and can forget to button up his shirt correctly. He has trouble getting in and out of the shower and needs to be reminded to wash. He has no issues with continence or in getting around the house.

**Cognitive impairment:** Mr Dunne’s short-term memory and concentration are moderately impaired. These difficulties were first recognised four years ago.

**Physical and mental health:**  Mr Dunne can forget he is not as mobile as he used to be and has had several falls in the past year but no serious injury. He regularly walks into the village which is some distance away and on a busy road and someone has to drop him home. He has hallucinations periodically which he finds confusing and distressing.

**Informal Support**: Mr Dunne is supported by his daughter who lives nearby. She spends several hours with Mr Dunne every day. She worries a lot about how they are going to cope in the future. She is taking medication for anxiety and depression and has a young family.

**Care preferences:** Mr Dunne is happy living at home but his daughter wants him to move to a nursing home.
